# Relative Value of Colchicum Root

**Published:** 1877-09

**Authors:** Theodore F. Beckert


### Selections.

---

THE RELATIVE VALUE OF COLCHICUM ROOT.—*By Theodore F. Beckert, Ph.G.*—This subject was suggested by several pharmacists, who of late have found it a difficult matter to obtain colchicum root which, on breaking, presented a clear white color. The recently imported article, as obtained from the wholesale druggists, consisted of tubers which had been sliced very irregularly. Out of a one pound lot not less than seven whole tubers were taken, the remainder varying from one-sixth to one-half inch in thickness. These pieces, when broken, presented quite a varied appearance, their color being all shades between white and black; and it was noticed that the lighter colored roots were mostly easy to break, and many of them of a mealy character, whereas the darker ones were difficult to break, and had a somewhat resinous appearance. A quantity of the root was broken piece by piece, and then separated into three grades, according to color, white, slate-colored, and brown or blackish, particular care being taken in the sorting. Upon weighing, it was found that the white root constituted only one-sixth, while the gray root comprised not quite two-sixths, and the black root a little over three-sixths of the article examined. These results also agree with the observations of several resident pharmacists.

The methods used to determine were as follows: Two troy-onces of each of the three grades of roots were exhausted by means of alcohol, yielding in each case about twelve fluid-ounces of tincture; these tinctures varied in color according to the grade of root used, that from the white root being lightest. This indicates the solubility in the alcohol of the foreign coloring matter present in the gray and black roots. In preparing these tinctures, care was taken to percolate them under as similar circumstances as possible.

The tinctures obtained were separately evaporated by means of a water bath; the residue was treated with distilled water, and poured upon a filter, in order to separate resinous matter; the filtrate was washed with slightly acidulated water until each filtrate measured 100 cc. Dilute sulphuric acid was used for acidulating the solutions, which were volumetrically treated with Mayer's solution, in quantities varying from 5 to 15 cc. In preliminary experiments the solutions were variously diluted, and it was observed that the results were very considerably influenced thereby, an observation previously made by Dragendorff. To serve as a basis for comparison, the experiments were afterwards made with solutions of uniform strength, as stated above, partly without any other addition, and partly as recommended by Dragendorff, after the addition of a concentrated solution of chloride of sodium, to increase the distinctness of the reaction. The three grades of the root required for 1 cc. respectively .0403, .0414 and .0462 of Mayer's solution.

Five troyounces of each of the roots were next exhausted by alcohol, percolation in each case being carried on until the liquid passed tasteless. The alcohol was evaporated, and the residues were treated with water, filtered and precipitated by a solution of tannin. These tannates of the white, gray and black roots, which, after having been dried, weighed respectively .32, .265 and .27 gram, were decomposed by oxide of lead, and then treated with alcohol, in order to separate colchicia. The three alcoholic solutions were carefully evaporated to dryness, then placed over sulphuric acid for several days, and then their weight taken; the product from the gray root weighing .115 gram, the black yielding .104 gram, while root was unfortunately lost.

I next obtained same colchicum root from Professor Maisch, which was not less than ten years old, it having been in his possession at least nine years. It had quite a handsome appearance, very little dark root being present, and in all respects was a much better looking article than that previously employed. Two troyounces of this root were treated as stated above, and an acid solution obtained, measuring 100 cc., and which, when treated with Mayer's test, in a similar manner as before, required .0300 for the precipitation of 1 cc.

The various results thus obtained are more concisely presented in the following table:

| | 1st grade or white root | 2d grade or gray root. | 3d grade or black root. | Very old root. |
|---|---|---|---|---|
| Mayer's solution necessary to precipitate 1 cc. of the solution....... | .0403 | .0414 | .0492 | .0300 |
| Percentage of alkaloid in air-dry root. | .205 | .210 | .219 | .152 |
| Tannate precipitate obtained from five troyounces of root........ | .320 | .265 | .270 | |
| Amount of crude colchicia obtained from the tannates............ | lost. | .115 | ,104 | |

By the above table it will be seen that the results obtained with tannin and by Mayer's solution do not agree as to the amount of colchicia indicated. This may be due to the slight solubility in water of the tannate of colchicia, as noticed by

Hiibler and others, and to the varying amount of water used in the last experiments. But the results seem to indicate that it apparently matters little whether the root has a white, gray or black color, but that the *age* is of primary importance, and none but a fresh-looking root should be purchased; if this is done I think no fault can be found as to the quality of the preparations made from it.

